# Effectiveness of Acupuncture in the Treatment of Hyperemesis Gravidarum: A Systematic Review and Meta-Analysis

**DOI:** 10.1155/2021/2731446

**Published:** 2021-07-27

**Authors:** Haizhen Lu, Chengwen Zheng, Yanmei Zhong, Linhao Cheng, Yi Zhou

**Affiliations:** ^1^School of Basic Medical Sciences, Chengdu University of Traditional Chinese Medicine, Chengdu, Sichuan 611137, China; ^2^School of Medical Information Engineering, Chengdu University of Traditional Chinese Medicine, Chengdu, Sichuan, China; ^3^School of Foreign Languages, Chengdu University of Traditional Chinese Medicine, Chengdu, Sichuan, China

## Abstract

**Background:**

Hyperemesis gravidarum (HG) is a common gastrointestinal disease afflicting gravidas. It usually results in hospital admission in early pregnancy.

**Objective:**

Through a meta-analysis, this study intended to explore acupuncture's clinical efficacy in treating HG.

**Materials and Methods:**

A comprehensive search of PubMed, the Cochrane Library, EMBASE, Web of Science, China National Knowledge Infrastructure (CNKI), Chinese Biological Medical (CBM), Wanfang Database, and China Science and Technology Journal (VIP) for published clinical randomized controlled trials (RCTs) of acupuncture for treating HG was conducted from the date of database creation to 20th January 2021. We also searched grey literature in four databases: Chinese Cochrane Center, Chinese Clinical Trial Registry, GreyNet International, and Open Grey from their inception to 20th January 2021. Two authors independently screened the literature, extracted data, and evaluated the quality of the literature with Cochrane Handbook 5.1.0 and Review Manager 5.2 software. Review Manager 5.2 and STATA 12.0 software were applied to analyze data. Heterogeneity analysis was performed by the Cochran Chi-square test and *I*^2^ statistic. Egger's tests together with funnel plots were used to identify publication bias.

**Results:**

A total of 16 trials covering 1043 gravidas were included. Compared with the conventional treatment, acupuncture had a significantly higher effective rate (OR: 8.11, 95% CI: 5.29∼12.43; *P* < 0.00001), a higher conversion rate of urine ketone (RR: 1.36, 95% CI: 1.15∼1.60; *P*=0.0003), an improvement rate of nausea and vomiting (OR: 26.44, 95% CI: 3.54∼197.31; *P*=0.001), and a relatively higher improvement rate of food intake (RR: 1.17, 95% CI: 1.01∼1.36; *P*=0.04). Acupuncture also shortened hospitalization time and manifested with a lower pregnancy termination rate and fewer adverse events. Nevertheless, no statistical variation in the improvement of nausea intensity, vomiting episodes, and lassitude symptom, recurrence rate, and serum potassium was observed.

**Conclusion:**

Our study suggested that acupuncture was effective in treating HG. However, as the potential inferior quality and underlying publication bias were found in the included studies, there is a need for more superior-quality RCTs to examine their effectiveness and safety. PROSPERO registration number: CRD42021232187.

## 1. Introduction

Hyperemesis gravidarum (HG) is generally regarded as severe nausea and vomiting during pregnancy (NVP); presently, there is no consensus definition towards it [1]. HG is generally defined as severe intractable vomiting, weight loss >5%, dehydration, ketonuria, malnutrition, and electrolyte imbalance [2, 3], whose incidence is about 1%. HG is usually responsible for hospitalization in early pregnancy [4], brings about serious physical and mental disorders [5–7], affects the postpartum period [6, 8], and poses an impact upon the quality of life [9].

At present, the etiology and the pathogenesis of HG are still unclear. According to contemporary medicine, the development of HG is correlated with enhanced human chorionic gonadotropin (HCG) levels, estrogen levels, neurological disorders, vitamin B deficiency, *Helicobacter pylori* infection [10, 11], weakened gastrointestinal motility, hypofunction of the adrenal cortex and anterior pituitary gland, and thyroid dysfunction, as well as genetics, mental and psychological state, and economic status [12–16].

Common clinical treatments involve intravenous rehydration, parenteral nutrition, correction of electrolyte disturbances, vitamin supplements, ginger, antiemetics, dopamine antagonists, serotonin 5-hydroxytryptamine Type 3 (5-HT3) receptor antagonists, *Helicobacter pylori* treatment, and intramuscular injection of vitamin B6, etc. [17–21]. However, due to the unsatisfactory efficacy of conventional treatments, adverse reactions, and teratogenic risks, people resort to complementary and alternative therapies [22].

As a complementary and alternative therapy, acupuncture is under practice for treating HG in many countries [18]. It has achieved certain therapeutic effects by stimulating acupoints. Acupuncture relieves nausea and vomiting to treat HG through the control and coordination of sympathetic and parasympathetic by activating the central structure and may also be pertinent to the regulation of neuroendocrine mechanisms [23]. However, according to guidelines, the effectiveness of acupuncture for HG is still controversial [24–26], which may be attributed to its different diagnostic and efficacy standards and lack of high-quality randomized controlled trials (RCTs). Hence, it is necessary to evaluate the efficacy of acupuncture for HG comprehensively and objectively.

Although there are different forms of acupuncture, such as auricular acupuncture, press needle (thumb-tack needle, a method of pressing very short needles into superficial subcutaneous) and hydroacupuncture (acupoint injection, a method of injecting medicine into acupoints), this study only focused on common forms of acupuncture (traditional acupuncture, body acupuncture, manual acupuncture, and electroacupuncture). Our objective is to systematically evaluate the efficacy of acupuncture for treating HG by unifying the inclusion criteria and screening out RCTs with common forms of acupuncture for HG patients, which may provide valuable evidence-based medical solutions for clinical medical performance.

## 2. Methods

PRISMA (Preferred Reporting Items for Systematic Reviews and Meta-Analyses) statement guidelines (Table S1) are conformed to in this meta-analysis and system review [27]. There is no systematic review protocol for this study, but we have registered this systematic review on PROSPERO: CRD42021232187.

### 2.1. Search Strategy

This meta-analysis is a literature review; hence, approval from the institutional review board is not compulsory.

Such databases as PubMed, EMBASE, Cochrane Library, Chinese Biological Medical (CBM), China National Knowledge Infrastructure (CNKI), China Science and Technology Journal (VIP), and Wanfang database were used for the retrieval from their inception to 20th January 2021. Besides, grey literature was retrieved from the Chinese Cochrane Center, Chinese Clinical Trial Registry, GreyNet International, and Open Grey from their inception to 20th January 2021. We retrieved reference works of included studies concomitantly. Chinese search terms include (“针刺” OR “针灸” OR “电针” OR“体针” OR “毫针”) AND (“妊娠剧吐” OR “妊娠恶阻”). The English search strategies including MeSH and text words are as follows, which may be slightly modified based on the requirements of different databases: hyperemesis gravidarum, pernicious vomiting of pregnancy, acupuncture, and acupuncture therapy. The retrieve was performed with the search strategies as the list: (Hyperemesis Gravidarum [MeSH] OR Pernicious Vomiting of Pregnancy [Title/Abstract] OR Pregnancy Pernicious Vomiting [Title/Abstract]) AND (Acupuncture [MeSH] OR Acupuncture Therapy [MeSH] OR Acupuncture Points [MeSH] OR Acupuncture Treatment *∗* [Title/Abstract] OR Electroacupuncture *∗* [Title/Abstract] OR Acupoint *∗* [Title/Abstract]) AND (randomized controlled trial [Publication Type] OR randomized [Title/Abstract] OR placebo [Title/Abstract]) (Table 1). The management of all references was achieved by Endnote X9.2 software.

### 2.2. Eligibility Criteria

#### 2.2.1. Inclusion Criteria

1. Type of participants: the subjects are patients diagnosed with HG (diagnosed by a clinician or using any recognized diagnostic criteria), regardless of ethnicity, country, age, and course of the disease.
2. Type of interventions: the treatment group received the common forms of acupuncture solely or combined with other treatments, regardless of acupoint selection, treatment frequency, or course. The control group adopted conventional symptomatic treatment, conventional medication, placebo, sham, or no treatment. The two groups could receive the same basic treatment.
3. Type of comparisons: the control group adopted conventional treatment, medication, placebo acupuncture, sham acupuncture, or no treatment.
4. Type of outcomes: the effective rate, the conversion rate of urine ketone, symptom improvement rate, serum potassium, hospital stay, pregnancy termination rate, adverse events, and recurrence.
5. Type of studies: the study type was confined to RCTs, and the qualified articles were limited to Chinese or English language (Table 2).

#### 2.2.2. Exclusion Criteria

1. Participants with serious organic diseases or medical diseases that can induce vomiting.
2. The treatment group received traditional Chinese medicine or other forms of acupuncture (such as auricular acupuncture, hydro-acupuncture and press needle). The control group was combined with acupuncture-related therapies.
3. We also excluded trials that simultaneously conducted acupuncture and acupuncture-related therapies (such as acupressure and moxibustion) in the treatment group where the mainstay of the acupuncture could not be identified.
4. Excluded reviews, theoretical discussion, case reports, animal experiments, crossover trials, and non-RCTs.
5. Excluded duplicate publications and studies with incomplete data.

### 2.3. Outcome Measurements

The results are mainly expressed by the effective rate: the number of cases (cured + markedly effective + improved)/total number of cases × 100%. The secondary outcomes are the conversion rate of urine ketone, symptoms improvement rate (nausea intensity, vomiting episodes, nausea and vomiting, food intake, and lassitude), serum potassium, length of hospital stay, and the rate of pregnancy termination, adverse events, and recurrence.

### 2.4. Study Selection

All papers were imported into the software Endnote X9.2 for management. Two authors (Haizhen Lu, Chengwen Zheng) independently screened the literature. Endnote X9.2 was used to sort all literature, and two authors manually put the documents with duplicate titles and abstracts into the “duplicate documents” folder for management. After excluding duplicates, by reviewing the title and abstract, studies that did not meet the criteria of the type of studies, participants, interventions, comparisons, and outcomes were removed. And then, studies that met the above eligibility criteria were covered after reviewing the full text. Any problems in the process of selecting the included studies should be resolved through discussion or deliberation with the third author (Yanmei Zhong).

### 2.5. Data Extraction

Data were extracted independently by two authors (Haizhen Lu, Chengwen Zheng) who checked the extracted data with each other. Any disagreements are resolved through conferencing or consulting with the third author (Yanmei Zhong). Available information was recorded by Microsoft Excel 2010 with standard designed forms, including baseline information (first author, study type, country, publication year, age, gestational age, sample, intervention of the treatment and control group, duration of treatment, and relevant outcomes) and outcomes (effective rate, conversion rate of urine ketone, improvement rate of nausea intensity, vomiting episodes, nausea and vomiting, food intake, and lassitude symptom, serum potassium, length of hospitalization, rate of pregnancy termination, adverse events, and recurrence).

### 2.6. Bias Risk Assessment

Two authors (Haizhen Lu, Chengwen Zheng) independently evaluated the quality of each study. The Cochrane Handbook 5.1.0 and RevMan 5.2 software were used to evaluate the quality of each of the studies, including bias risk in the following items: random sequence generation; whether to assign hiding; whether to blindly perform blinding to participants and researchers; whether to use blinding for result measurement; whether the outcome data is complete, whether to report selectively; other bias. The risk of bias was ranked as “low,” “high,” or “unclear.” Any problems were resolved through conferencing or consulting with the third author (Yanmei Zhong).

### 2.7. Data Analysis

The combined risk ratio (RR) and odds ratio (OR) were utilized for evaluating the dichotomous outcomes at the 95% confidence interval (95% CI); the continuous outcomes were represented by the mean difference (MD) of 95% CI. Study heterogeneity was expressed as the Cochran Chi-square test and *I*^2^ statistic. *I*^2^ > 50% indicated significant heterogeneity. To summarize the results, the random-effects and fixed-effects models were applied, respectively, when *I*^2^ > 50% and *I*^2^ < 50%. A concomitant sensitivity analysis was performed for identifying the potential origin of inhomogeneity. Then, studies that caused marked inhomogeneity were deleted, and the remaining studies were re-meta-analyzed to adjust. If there is no substantial difference between the results before and after the adjustment, the robustness of our meta-analysis will be confirmed [28]. The conversion of effect mode conducted a sensitivity analysis on outcomes with small heterogeneity to assess whether the result is robust. Egger's tests together with funnel plots were performed to identify whether there was publication bias. Statistical analysis was conducted by RevMan5.2 and STATA 15.0.

## 3. Results

### 3.1. Study Selection

**Figure 1:**
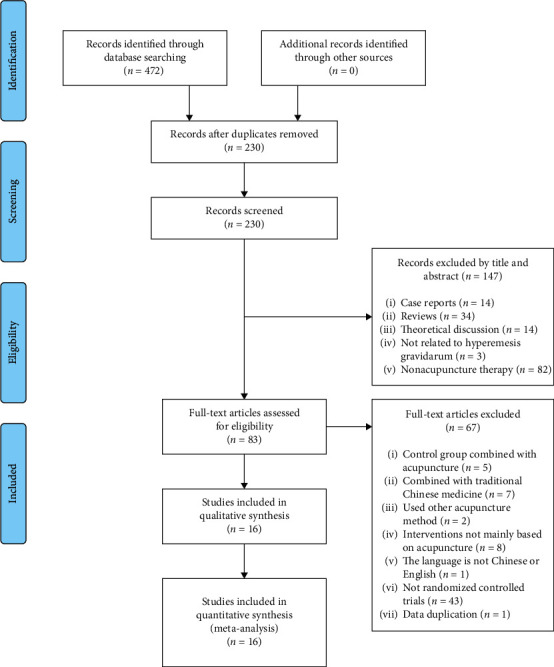
PRISMA flow diagram.

According to the searching strategy, 472 papers were retrieved from the inception of databases to 20th January 2021. And during the search of grey literature, no suitable or valuable literature and information was obtained. After deduplication, a total of 230 records were identified. Then, 147 records were removed after reading titles and abstracts. After reading all the full text, 67 papers out of residual 83 articles were removed from the analysis due to the following reasons: five papers were combined with acupuncture in the control group, seven were combined with traditional Chinese medicine, two used other acupuncture methods, eight articles were not mainly based on acupuncture, one was not in Chinese or English, forty-three papers were not RCTs, and one was with data duplication. In the end, 16 studies were selected to be incorporated in this meta-analysis, as illustrated in Figure 1 [18, 19, 29–42].

### 3.2. Study Characteristics

Overall, 14 studies are in Chinese and two in English. All studies reported comparable or nonsignificant differences in general information such as age and gestation period between intervention groups. Among them, there were one four-arm trial [18] and two three-arm trials [41, 42], and the rest were two-arm trials. The sample size of each group ranged from 8 to 50 cases. The treatment group and the control group involved seven therapeutic methods: acupuncture, acupuncture placebo, moxibustion, acupoint sticking (a method of applying ointments on acupoints), acupressure, intravenous rehydration, and conventional medicine. The shortest duration of treatment was 3 days and the longest was 14 days, but no specific duration was reported in three studies [36, 38, 39]. No follow-up was recorded. Fifteen studies reported the effective rate [18, 29–42], 3 studies presented the conversion rate of urinary ketone [29, 30, 40], 1 study mentioned the improvement rate of nausea intensity and vomiting episode [19], 2 studies referred to the improvement rate of nausea and vomiting [30, 40], 3 studies mentioned the improvement of food intake [19, 30, 40], 2 studies were related to improvement rate of lassitude symptom [30, 40], 2 studies evaluated serum potassium [29, 31], 2 studies reported length of hospital stay [29, 35], 2 studies stated pregnancy termination rate [29, 31], 1 study referred to recurrence rate [35], and 1 study mentioned adverse events [38]. Tables 3 and 4 list the included study's features.

### 3.3. Bias Risk Assessment

**Figure 2:**
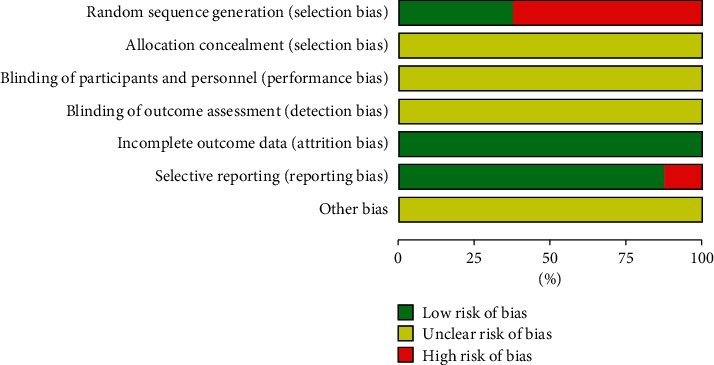
Diagram of the bias risk.

**Figure 3:**
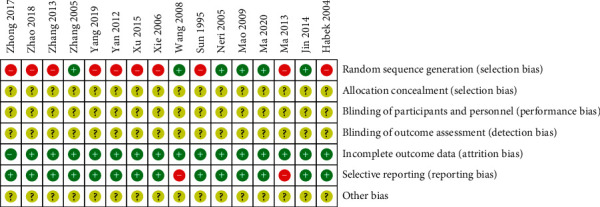
Summarized bias risk.

The trials of 16 studies selected were all randomized controlled. As identified by the corresponding Cochrane tool, all studies mentioned randomization, of which 6 studies [18, 32, 35, 37–39] did not report the specific method of randomization, and 4 studies [33, 34, 36, 40] used the higher risk of random methods, such as the order of registration or treatment sequence. And 6 studies [19, 29–31, 41, 42] applied the random number table method or computer random number method to generate random sequences. None of the studies mentioned the application of allocation concealment information. As for the blinding method, only one study [40] mentioned “single blinding” but the detailed operation description was absent. No study mentioned evaluator blinding. The bias risk of all trials was rated low regarding outcome completeness. Two studies [31, 32] had selective reports. Other biases were classified as unclear due to insufficient information. In general, included RCTs were of low quality. Figures 2 and 3 summarize the quality evaluation of eligible studies.

### 3.4. Results of Meta-Analysis

#### 3.4.1. Effective Rate

**Figure 4:**
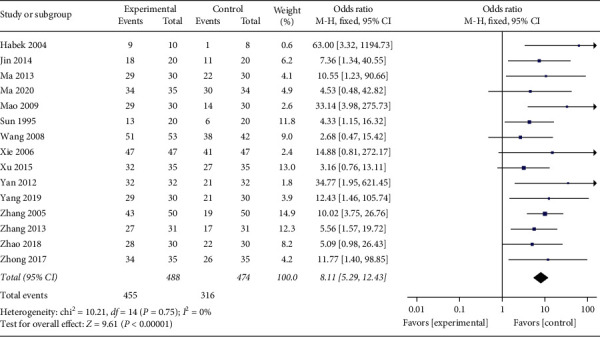
Outcomes of the meta-analysis for the influence of effective rate.

The effective rate is the main outcome of this study. Fifteen studies [18, 29–42] compared the effective rate at the end of treatment. And 15 trials reported data on 962 participants. The between-study *I*^2^ statistical heterogeneity was 0%, and the *P* value of the *χ*2 test was 0.75 (*P* > 0.1). No marked heterogeneity existed between the studies, so the fixed-effects model was selected for analysis. The results showed that the effective percentage of the acupuncture set was significantly higher than other active treatments in the control group (OR: 8.11, 95% CI: 5.29∼12.43; *P* < 0.00001). The result of *P* < 0.00001 indicated an evident group difference (Figure 4). There was no significant heterogeneity between the studies, so no subgroup analysis was performed

#### 3.4.2. Conversion Rate of Urine Ketone

**Figure 5:**
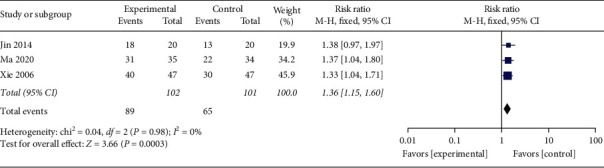
Resultants of a meta-analysis for the affection of the conversion rate of urine ketone.

Three studies [29, 30, 40] compared the conversion rate of urine ketone after treatment with the control group. And 203 participants were included in 3 studies. With no obvious heterogeneity between the studies (*P*=0.98, *I*^2^ = 0%), the fixed-effects model was employed for analysis. The results implied that, compared with the control group, the conversion rate of urine ketone was higher in the acupuncture group (Figure 5, RR: 1.36, 95% CI: 1.15∼1.60; *P*=0.0003). Since *P*=0.0003, a clear difference between the two groups was present.

#### 3.4.3. Improvement Rate of Nausea Intensity

One study [19] reported an improvement in the nausea intensity after the treatment. The random effect model implied the absence of any statistical difference between the acupuncture set and the conventional medication set in terms of nausea intensity improvement (RR: 1.40, 95% CI: 0.79∼2.49; *P*=0.25). Since *P*=0.25, no evident difference could be seen between the two groups.

#### 3.4.4. Improvement Rate of Vomiting Episodes

One study [19] reported an improvement rate of vomiting episodes before and after treatment. The random-effects model revealed the nonexistence of a statistically significant group difference (RR: 1.51, 95% CI: 0.92∼2.48; *P*=0.10).

#### 3.4.5. Improvement Rate of Nausea and Vomiting Symptom

**Figure 6:**
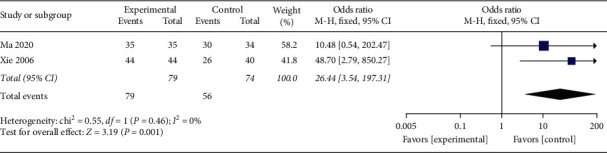
The meta-analysis results of the improving rate effects of nausea and vomiting symptoms.

Two studies [30, 40] reported the improvement rate of nausea and vomiting symptoms with 153 participants in total. The heterogeneity test showed good heterogeneity (*P*=0.46, *I*^2^ = 0%), using fixed-effects model. The combined effect amount results revealed that the improvement rate of nausea and vomiting symptoms in the acupuncture setting is markedly higher than the control set (Figure 6, OR: 26.44, 95% CI: 3.54∼197.31; *P*=0.001). Since *P*=0.001, the divergence between both sets was statistically distinct.

#### 3.4.6. Improvement Rate of Food Intake

**Figure 7:**
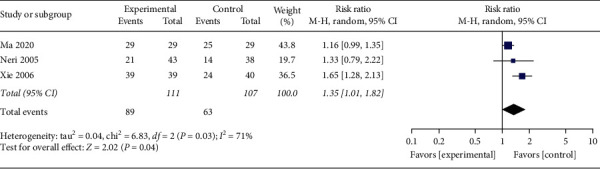
The meta-analysis results of improvement rate effects of food intake.

**Figure 8:**
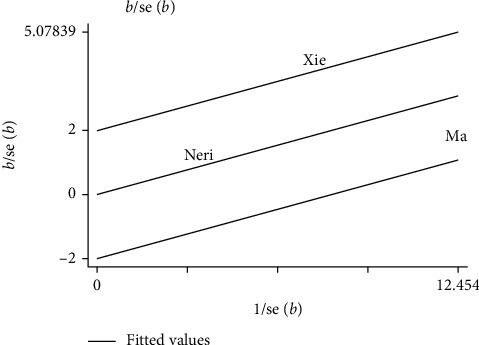
Sensitivity of the improvement rate of food intake (Galbraith).

**Figure 9:**
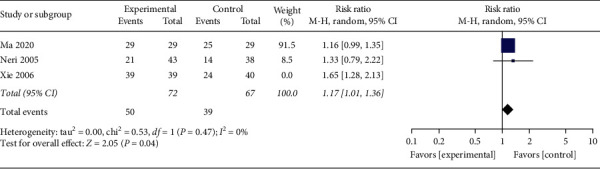
The re-meta-analysis results of the improving rate effects of food intake.

Three studies [19, 30, 40] reported the improvement rate of food intake after the treatment. A total of 3 trials evaluated 218 enrolled participants. Meta-analysis with stochastic effect mode implied that the improvement rate of food intake was higher in the acupuncture group (Figure 7, RR: 1.35, 95% CI: 1.01∼1.82; *P*=0.04) with a moderate study heterogeneity (*P*=0.03, *I*^2^ = 71%). Therefore, to further explore possible sources of heterogeneity, sensitivity was analyzed. Galbraith diagram showed 1 study: Xie [40] might have a substantial impact on inhomogeneity (Figure 8). After exclusion of this study, we got no obvious heterogeneity (Figure 9, *I*^2^ = 0%, *P*=0.47). Combining the results of the two studies, the revised correction result indicated the improvement rate of food intake in the acupuncture set was still higher than the control set (Figure 9, RR: 1.17, 95% CI: 1.01∼1.36; *P*=0.04); the meta-analysis results were robust.

#### 3.4.7. Improvement Rate of Lassitude Symptom

**Figure 10:**
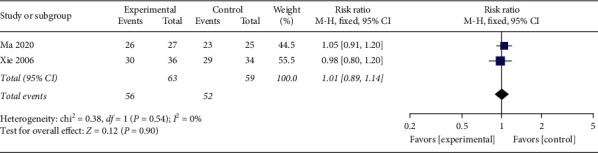
The meta-analysis outcomes of the amelioration rate of lassitude symptom.

Two studies [30, 40] reported an improvement rate of lassitude symptom of 122 participants; *I*^2^ = 0% and *P*=0.54 suggested no obvious heterogeneity between the studies. Using the fixed-effects model, the pooled data showed that no marked difference existed between the acupuncture set and the control set in the improvement rate of lassitude symptom (Figure 10, RR: 1.01; 95% CI: 0.89∼1.14; *P*=0.90). Since *P*=0.90, the divergence between both groups was not statistically distinct.

#### 3.4.8. Serum Potassium

**Figure 11:**
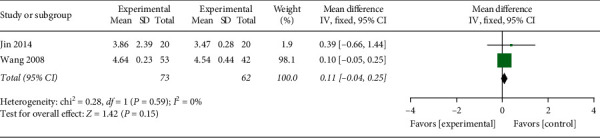
The meta-analysis outcome of the influence study of serum potassium.

Two studies [29, 31] reported the serum potassium of 135 participants. With no obvious heterogeneity being seen (*P*=0.59, *I*^2^ = 0%), the fixed-effects model was run for analysis. According to the results, *P*=0.15 indicated no marked divergence between the two groups in improving serum potassium (Figure 11, WMD: 0.11, 95% CI: −0.04∼0.25; *P*=0.15).

#### 3.4.9. Length of Hospital Stay

**Figure 12:**
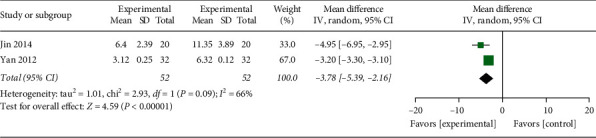
The meta-analysis outcome of the interactions of the length of hospital stay.

Two studies [29, 35] were found to report the length of hospital stay in the therapeutic team and the control team. There was a moderate study heterogeneity (*P*=0.09, *I*^2^ = 66%). Consequently, an analysis was conducted via the random-effects model. The results exhibited that *P* < 0.00001, a remarkable difference between groups was observed, indicating that the hospital stays in the acupuncture group were less than conventional treatments in the control group (Figure 12, WMD: −3.78, 95% CI: −5.39∼−2.16; *P* < 0.00001), and the variance between both sets was statistically significant (*P* < 0.00001).

#### 3.4.10. Pregnancy Termination Rate

**Figure 13:**
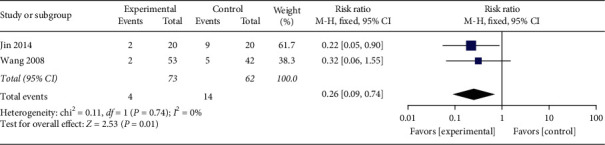
The meta-analysis outcome of pregnancy termination incidence.

Two studies [29, 31] reported a pregnancy termination rate of 135 participants. There was no obvious heterogeneity (*P*=0.74, *I*^2^ = 0%), so the fixed-effects model was used to perform further analysis. The results demonstrated that the pregnancy termination rate in the acupuncture group was lower than conventional interventions in controls (Figure 13, RR: 0.26, 95% CI: 0.09∼0.74; *P*=0.01), showing a statistically significant difference (*P*=0.01).

#### 3.4.11. Recurrence Rate

Only one study [35] amplified the relapse rates of the therapeutic set and the control set after the whole therapy. The random-effects model illustrated that *P*=0.14, and the between-study difference was not statistically distinct, so the acupuncture set did not differ significantly from the control set concerning the recurrence rate (RR: 0.11, 95% CI: 0.01∼1.98; *P*=0.14).

#### 3.4.12. Adverse Events Rate

One study [38] was found to report adverse events. In the acupuncture set, there was one skin rash case; in the control set, there were two cases of skin rash, three of dizziness, and three of facial flushing. Compared with the control set, the random-effects model suggested that the incidence of adverse events in the acupuncture group was lower (RR: 0.13, 95% CI: 0.02∼0.94; *P*=0.04). Since *P*=0.04 (*P* < 0.05), there was a marked divergence between both sets.

### 3.5. Publication Bias

**Figure 14:**
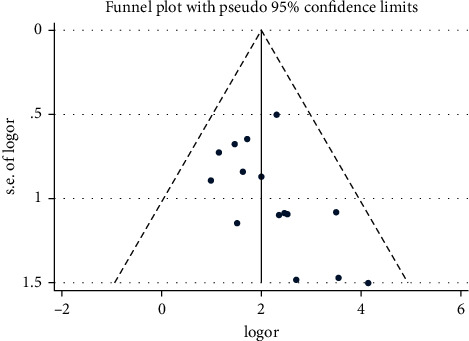
Publication bias of the effective rate.

According to the funnel plot, there was no significant asymmetry for the effective rate (Figure 14). In Egger's test, the *P* value of the effective rate of 0.08 (*P* > 0.05) and the conversion rate of urine ketone of 0.322 (*P* > 0.05) indicated no publication bias in the meta-analysis of the effective rate and the urinary ketone conversion rate. Because the number of included studies for other outcomes was too small (*n* < 5), the funnel plot was not performed.

### 3.6. Sensitivity Analysis

For outcomes without obvious heterogeneity, such as the effective rate, conversion rate of urine ketone, symptom improvement rate of nausea and vomiting, and lassitude, serum potassium, and pregnancy termination rate, sensitivity analyses were carried out through the method of conversion effect model. For studies with obvious heterogeneity, such as the improvement rate of food intake, the sensitivity analysis was performed by excluding individual studies. The same effects indicated the robustness of the combined result.

## 4. Discussion

Studies meeting inclusion criteria, from 1995 to 2020, have been incorporated into this meta-analysis to make evaluations on the outcome of acupuncture in the treatment of HG. Totally, 16 studies and 1043 patients (531 vs 512) were covered. According to this study, acupuncture was more effective than the conventional treatment of HG; it could better promote the conversion of the urinary ketone to improve ketonuria. In terms of symptoms, acupuncture seems to be more effective in reducing nausea and vomiting and increasing food intake. However, one study showed that there was no difference between acupuncture and conventional medication (metoclopramide infusion and oral vitamin B12 complex) in reducing the intensity of nausea and reducing the onset of vomiting. The reasons for the different results may be caused by the different focus of the outcomes, evaluation criteria, and intervention plans. In addition, the possibility could not be ruled out whether acupuncture and the medication (metoclopramide and vitamin B12 complex) had no difference in efficacy because the relationship between acupuncture and the medicine was unclear. However, it should also be noted that because the number of studies on the outcomes of symptoms improvement is too small (*n* < 3) and the sample size is small, the role of acupuncture in improving these symptoms needs more trials to verify. Furthermore, this meta-analysis uncovered that acupuncture seems to shorten the hospitalization time, but since only two studies mentioned this result, and the heterogeneity is moderate, which may be related to the severity of the disease, so whether acupuncture can shorten hospital stay needs further research. Besides, there were data showing that acupuncture manifested lower occurrence of adverse events and pregnancy termination. But also, because of the small number of included studies and the low quality of the evidence, further studies on the safety of acupuncture treatment are needed in the future. However, acupuncture does not seem to have any advantages in reducing recurrence and improving serum potassium and symptoms of lassitude. Due to the small number of included studies of secondary outcomes (*n* < 5), we must be cautious about their role in acupuncture. With the increase and improvement of future trials, the results may be reversed. In general, due to the low quality of the included studies and the potential bias risk, low evidence supports the positive effects of acupuncture in HG.

As one of the complementary therapies, acupuncture is common in the treatment of HG. Through the stimulation of meridians and acupuncture points, acupuncture can regulate qi-blood as well as yin-yang and improve the function of the viscera [43]. Although similar treatments, such as acupressure and hydro-acupuncture, are commonly used in HG, acupuncture still has its unique advantages. Acupuncture outperforms acupressure in the onset and duration of treatment. Acupressure requires prolonged (at least 8–12 hours) and correct use of the wristband to maintain curative effect [44]. Carlsson et al. observed that acupuncture could relieve vomiting in the treatment of HG within a few minutes, which may be associated with some neural substrate [45]. Besides, acupressure often solely selects Neiguan (PC6), the commonly used acupoint in treating HG, whereas acupuncture is more flexible, dialectically selecting acupoints according to individual conditions and the holistic situation. Moreover, Habek et al. found that acupuncture was superior to acupressure in treating HG, probably owing to the milder stimulation of acupressure and faster and more intense neurophysiological effects yielded by acupuncture [18]. In addition, acupuncture is often referred to as mind-body therapy. It has favorable effects on both mental and gastrointestinal psychosomatic disorders through the regulation of the autonomic nervous system [46–48]. The role of acupuncture in reducing the anxiety and depression scores in patients with nausea during pregnancy has been reported previously [49]. As for hydroacupuncture therapy, although its efficacy on HG has been demonstrated to some extent [22], further research is required because of its considerable stimulation, significant pain, unclear adverse effects, and action mechanism. The mechanisms of acupuncture in the treatment of HG are gradually being explored and currently focus its antiemetic and gastrointestinal function. Many experiments have demonstrated the influence of acupuncture on the endogenous opioid system [50] as well as 5-hydroxytryptamine transmission [51] by activating 5-hydroxytryptamine and noradrenergic fibers, thereby affecting the afferent stimulation of the central nervous system to the vomiting center and reducing nausea and vomiting. In addition, it can not only regulate gastrointestinal motility by stimulating the reflexes of the vagus and sympathetic nerves [52, 53] but also affect gastric emptying via somatic visceral reflexes [54, 55] to modify nausea and vomiting. What is more, recent studies have uncovered that the use of acupuncture during pregnancy may be a safe treatment modality to mitigate discomfort [56, 57], with no report of severe negative events on mothers or newborns [58].

There are some strengths in this study. At present, there are systematic reviews on all interventions of HG, and the latest one only included 3 acupuncture trials published in 2018 [59]. But as far as we know, this study is the first systematic review and meta-analysis to comprehensively evaluate the effectiveness of acupuncture for HG without geographical restrictions. We mainly focus on the role of common forms of acupuncture, which reduces the possible variability of other acupuncture forms on the results and more accurately evaluates the effects of acupuncture in HG. We have also included the outcome indicators for acupuncture in HG as comprehensively as possible. Although the number of studies included in the secondary outcomes is small, we hope that this will provide more possibilities and references for future clinical research and practice. At present, acupuncture is a commonly used complementary therapy for HG. Our study specifically addresses this issue and supports that acupuncture may be used as a complementary and alternative therapy for HG.

In short, acupuncture is an effective approach for HG and has a great potential to mitigate ketonuria, ease nausea and vomiting, increase food intake, and shorten the hospital stay. There is some evidence that acupuncture may be utilized as a supplement and alternative to HG. However, the poor quality of the incorporated studies and potential publication bias can exert a certain impact on the results. For the better clinical application and promotion of acupuncture in the treatment of HG, more large-scale samples, multi-center, and top-quality RCTs are needed urgently. In the future, it is necessary to further study the relationship between acupuncture and conventional medication and acupuncture placebo to obtain the best clinical solution.

## 5. Limitations

This meta-analysis has demonstrated that acupuncture possesses favorable effects on HG. However, several limitations must be acknowledged. Firstly, 14 of the 16 included studies are Chinese, which tends to induce publication bias. Secondly, most of the included RCTs are small samples, and the number of studies included in the outcomes is small. Thirdly, since the particularity of acupuncture therapy, there are certain difficulties in the study of allocation hiding and blinding and there is a possibility of bias. The poor methodological quality, the flawed design of the incorporated research, and potential bias may disturb the accuracy of the results. Fourthly, the lack of uniformity in diagnostic and efficacy criteria and incomprehensive outcome indicators in the included studies may affect the precision of assessing the effectiveness of acupuncture treatment. Fifthly, due to the significant differences in acupoint selection and acupuncture stimulation, it is not conducive to unified data analysis. Sixthly, only one study [38] mentioned adverse event reports, and the safety reports were insufficient. Lastly, all studies were short-term efficacy evaluations, with no follow-up, and further research should be conducted to examine whether acupuncture has sustained effects on HG.

## Figures and Tables

**Table 1:** PubMed search strategy.

No.	Searches
#1	Hyperemesis Gravidarum [MeSH]
#2	Pernicious Vomiting of Pregnancy [title/abstract]
#3	Pregnancy Pernicious Vomiting [title/abstract]
#4	#1 OR #2 OR #3
#5	Acupuncture [MeSH]
#6	Acupuncture Therapy [MeSH]
#7	Acupuncture Points [MeSH]
#8	#5 OR #6 OR #7
#9	Acupuncture [title/abstract]
#10	Acupuncture treatment ^∗^ [title/abstract]
#11	Acupuncture therap ^∗^ [title/abstract]
#12	Pharmacopuncture [title/abstract]
#13	Pharmacoacupuncture treat ^∗^ [title/abstract]
#14	Electroacupunctur ^∗^ [title/abstract]
#15	Electro-acupunctur ^∗^ [title/abstract]
#16	Acupoint ^∗^ [title/abstract]
#17	Acupotom ^∗^ [title/abstract]
#18	Point^∗^, Acupuncture [title/abstract]
#19	Meridian ^∗^ [title/abstract]
#20	Moxibustion [title/abstract]
#21	#9 OR #10 OR #11 OR #12 OR #13 OR #14 OR #15 OR #16 OR #17 OR #18 OR #19 OR #20
#22	#8 OR #21
#23	Randomized controlled trial [publication type]
#24	Randomized [title/abstract]
#25	Placebo [title/abstract]
#26	#23 OR #24 OR #25
#27	#4 AND #22 AND #26

**Table 2:** Statement of participants, interventions, comparisons, outcomes, and study design (PICOS).

Participants (*P*)	Patients diagnosed with HG.

Interventions (*I*)	The treatment group received the common forms of acupuncture solely or combined with other treatments, regardless of acupoint selection, treatment frequency, or course.
	The control group adopted conventional symptomatic treatment, conventional medication, placebo, sham, or no treatment.
	The two groups could receive the same basic treatment.

Comparisons (*C*)	The control group adopted conventional treatment, medication, placebo acupuncture, sham acupuncture, or no treatment.

Outcomes (*O*)	The effective rate, conversion rate of urine ketone, symptom improvement rate, serum potassium, hospital stay, pregnancy termination rate, adverse events, and recurrence.

Study design (*S*)	RCT.

**Table 3:** Characteristics of included studies (1).

Study	Year	Country	Study period	Age (years) (mean ± SD)		Gestational age (weeks) (mean ± SD)	
				*T*	*C*	*T*	*C*
Habek et al. [18]	2004	Croatia	—	20.4 ± 4.7	20.8 ± 4.1	7 (6–9)^∗^	8 (7–12)^∗^
Neri et al. [19]	2005	Italy	2001.5–2002.7	—	—	—	—
Jin and Hu [29]	2014	China	2009.10–2013.10	25.09 ± 3.42	26.03 ± 3.19	9.03 ± 2.15	8.98 ± 2.28
Ma et al. [30]	2020	China	2013.10–2017.10	31.14 ± 4.06	31.36 ± 4.24	8.64 ± 1.22	8.36 ± 1.31
Wang [31]	2008	China	2005.1–2006.1	27 ± 3	27 ± 4	—	—
Ma and Meng [32]	2013	China	2010–2012	26.30		—	—
Zhao and Qiao [33]	2018	China	2015.4–2017.11	26 ± 3	26 ± 3	8.33 ± 1.94	8.03 ± 1.63
Zhang [34]	2013	China	2008.1–2012.12	28.58 ± 4.57	28.68 ± 3.76	8.90 ± 1.66	8.97 ± 1.58
Yan et al. [35]	2012	China	2008.1–2009.6	28.09 ± 5.78	28.03 ± 6.25	9.08 ± 2.44	8.95 ± 2.58
Xu et al. [36]	2015	China	2013.1–2014.10	(20–35)^#^		(4–12)^#^	
Sun and Cui [37]	1995	China	1992.1–1994.12	(22–34)^#^		—	—
Yang [38]	2019	China	2016.11–2018.11	31.56 ± 6.25	31.42 ± 6.37	8.52 ± 3.62	8.86 ± 3.57
Zhong [39]	2017	China	2015.9–2016.9	28.35 ± 2.76	27.93 ± 2.47	—	—
Xie [40]	2006	China	2004.1–2005.5	27.25 ± 3.35	27.46 ± 3.29	—	—
Mao and Liang [41]	2009	China	2001.1–2008.12	28.23 ± 4.73	28.63 ± 4.86	8.30 ± 1.60	8.33 ± 1.58
Zhang [42]	2005	China	1999.3	—	—	—	—

Abbreviation: SD: standard deviation; *T*: treatment group; *C*: control group; ^∗^: figures are median and range; ^#^: figures are a range.

**Table 4:** Characteristics of included studies (2).

Study	Intervention		Number of patients (*T*/*C*)	Intervention period	Relevant outcomes	Study type
	*T*	*C*				
Habek et al. [18]	AP	Placebo acupuncture	18 (10/8)	Over 7 days	②	RCT
Neri et al. [19]	AP + acupressure	Metoclopramide Infusion + oral vitamin B12 complex 30 mg/day	81 (43/38)	14 days	④⑩⑪	RCT
Jin and Hu [29]	AP + ①	①	40 (20/20)	Lasted for 5 days	②③⑦⑧⑨	RCT
Ma et al. [30]	AP + ①	①	72 (35/34)	Lasted for 7 days	②③④⑤⑥	RCT
Wang [31]	AP	①	95 (53/42)	Lasted for 6 days	②⑧⑨	RCT
Ma and Meng [32]	AP + TNA	TNA	60 (30/30)	Lasted for 5 days	②	RCT
Zhao and Qiao [33]	AP + acupoint sticking	①	60 (30/30)	Lasted for up to 7 days	②	RCT
Zhang [34]	AP + ① + psychological counseling	①	62 (31/31)	Lasted for 7 days	②	RCT
Yan et al. [35]	AP + ①	①	64 (32/32)	(3.12 ± 0.25) days/(6.32 ± 0.12) days	②⑦⑬	RCT
Xu et al. [36]	AP + moxibustion + ①	①	70 (35/35)	Unknown	②	RCT
Sun and Cui [37]	AP + ①	①	40 (20/20)	Lasted for 3 days	②	RCT
Yang [38]	AP + ①	①	60 (30/30)	Unknown	②⑫	RCT
Zhong [39]	AP + ①	①	70 (35/35)	Unknown	②	RCT
Xie [40]	AP + ①	①	94 (47/47)	Lasted for 10 days	②③④⑤⑥	RCT
Mao and Liang. [41]	AP + ①	① + oral luminal 30 mg, tid	60 (30/30)	Lasted for 7 days	②	RCT
Zhang [42]	AP + moxibustion	① + oral luminal 30 mg, tid	100 (50/50)	14 days	②	RCT

Abbreviation: *T*: treatment group; *C*: control group; RCT: randomized controlled trial; AP: acupuncture; TNA: total nutrient admixture; tid: three times a day; ①: symptomatic rehydration support treatment; ②: effective rate; ③: conversion rate of urine ketone; ④: improvement rate of food intake; ⑤: improvement rate of lassitude symptom; ⑥: improvement rate of nausea and vomiting symptom; ⑦:length of hospital stay; ⑧: serum potassium; ⑨: pregnancy termination rate; ⑩: improvement rate of nausea intensity; ⑪: improvement rate of vomiting episodes; ⑫: adverse events rate; ⑬: recurrence rate.

## Data Availability

The data used to support the findings of this study are included within the article. Also, the findings of this study are available from the corresponding author upon request.

## Supplementary Materials

Supplementary Materials Table S1: the checklist of PRISMA guidelines.
